# Apoptosis and metastasis inhibitory potential of pineapple vinegar against mouse mammary gland cells in vitro and in vivo

**DOI:** 10.1186/s12986-019-0380-5

**Published:** 2019-07-26

**Authors:** Nurul Elyani Mohamad, Nadiah Abu, Swee Keong Yeap, Kian Lam Lim, Muhammad Firdaus Romli, Shaiful Adzni Sharifuddin, Kamariah Long, Noorjahan Banu Alitheen

**Affiliations:** 1 Department of Cell and Molecular Biology, Faculty of Biotechnology and Biomolecular Science, Universiti Putra Malaysia (UPM), 43400 Serdang, Selangor Malaysia; 2 UKM Molecular Biology Institute (UMBI), UKM Medical Centre, Jalan Yaa’cob Latiff, Bandar Tun Razak, 56000 Cheras, Kuala Lumpur Malaysia; 3 China-ASEAN College of Marine Sciences, Xiamen University Malaysia, Jalan Sunsuria, Bandar Sunsuria, 43900 Sepang, Selangor Malaysia; 4 Faculty of Medicine and Health Sciences, Universiti Tunku Abdul Rahman, Sungai Long Campus, Jalan Sungai Long, Bandar Sungai Long, Cheras, 43000 Kajang, Selangor Malaysia; 5 Biotechnology Research Centre, Malaysian Agricultural Research and Development Institute (MARDI), 43400 Serdang, Selangor Malaysia; 6 Institute of Bioscience, Universiti Putra Malaysia (UPM), 43400 Serdang, Selangor Malaysia

**Keywords:** Mammary gland cancer, Pineapple vinegar, In vitro, In vivo

## Abstract

**Background:**

Plant-based food medicine and functional foods have been consumed extensively due to their bioactive substances and health-beneficial effects. Vinegar is one of them due to its bioactivities, which confers benefits on human body. Our previous study has produced pineapple vinegar that is rich in gallic acid and caffeic acid via 2 steps fermentation. There are many evidences that show the effectiveness of these resources in inhibiting the proliferation and metastasis of the cancer cells through several mechanisms.

**Methods:**

Freeze-dried pineapple vinegar was evaluated for its in vitro apoptosis and metastasis inhibitory potential using MTT, cell cycle, Annexin V and scratch assays. The in vivo test using BALB/c mice challenged with 4 T1 cells was further investigated by pre-treating the mice with 0.08 or 2 ml/kg body weight of freshly-prepared pineapple vinegar for 28 days. The tumor weight, apoptotic state of cells in tumor, metastasis and immune response of the untreated and pineapple vinegar treatment group were evaluated and compared.

**Results:**

From the in vitro study, an IC_50_ value of 0.25 mg/mL after 48 h of treatment was established. Annexin V/PI and scratch closure assays showed that pineapple vinegar induced 70% of cell population to undergo apoptosis and inhibited 30% of wound closure of 4 T1 cells. High concentration of pineapple vinegar (2 ml/kg body weight) led to the reduction of tumor weight and volume by 45%as compared to the untreated 4 T1-challenged mice. This effect might have been contributed by the increase of T cell and NK cells population associated with the overexpression of IL-2 andIFN-γ cytokines and splenocyte cytotoxicity. Furthermore, fewer instances of metastasis events were recorded in the pineapple vinegar treatment group and this could be explained by the downregulation of inflammation related genes (iNOS, NF-kB and COX2), metastasis related genes (iCAM, VEGF and MMP9) and angeogenesis related genes (CD26, TIMP1, HGF, MMP3, IGFBP-1 and IGFBP-2).

**Conclusion:**

The ability of pineapple vinegar to delay cancer progression portrayed its potential as chemopreventive dietry intervention for cancer therapy.

**Electronic supplementary material:**

The online version of this article (10.1186/s12986-019-0380-5) contains supplementary material, which is available to authorized users.

## Background

Cancer occurs when the growth of tissues is uncontrollable due to the unchecked proliferation of abnormal cells with the ability to invade other tissues [1]. Even though the numbers of cases have dropped each year, cancer is still one of the leading causes of death worldwide with breast cancer as the most common type of cancer in women [2]. Many chemotherapy and hormone-therapy drugs have been developed to cure or delay the progression of cancer; however,these drugs also bring about detrimental side effects [3]. Thus, the discovery of a potential chemo-preventive dietary intervention is welcomed [4].

Plants have the potential to rival the conventional treatments in treating cancer as they contain active compounds, which will work well as therapeutic agents andthere are vast historical records detailing the use of plants preparation in folk medicine [5, 6]. The ability of the natural resources to act as the chemo-preventive agents without harming the healthy tissues opens up a new field to be researched on [7]. The anti-inflammatory and anti-cancer effects of some bioactive compounds present in plants were identified and proven clinically [8]. Besides that, there have been initiatives to process them into supplements and health drinks, which will enable consumers to include them in their daily diet [9].

Pineapple (*Ananas comosus*) has been utilized clinically in different situations such as to treat burn, smoothen the bowel movement, and improve the immune system [10]. On the other hand, vinegar has been consumed for ages and its properties as anti-inflammation, anti-glycemic, and anti-hypertensive are extensively well known [11]. In the previous study, it was reported that pineapple vinegar was able to reverse the liver damage caused by an overdose of paracetamol in mice due to its anti-oxidant and anti-inflammatory effects [12]. To date, some studies have proven the in vitro and in vivo anti-tumor effect of several types of vinegars [13–15]. Nevertheless, no research has yet to be carried out to evaluate the possibility of using pineapple vinegar for cancer treatment, especially on breast cancer. Previously, we have reported that pineapple vinegar was rich in gallic acid, caffeic acid and several other phenolic acids [12]. There are many reports that have indicated the role of phenolic acids as onco-protective agent [16–18]. Thus, the in vitro study on the cytotoxic effect of pineapple vinegar on 4 T1 breast cancer cell was performed. Since positive results were observed, the pineapple vinegar was further investigated in vivo on the murine model. Through this study, the potential of pineapple vinegar as chemopreventive and dietry intervention for cancer therapy was recognized.

## Methods

### Preparation of pineapple vinegar

Pineapple vinegar was prepared according to the previous study [12]. In brief, pineapple juice underwent double fermentation process, first by anerobic fermentation using *Saccharomyces ceverisae 7013 INRA* to produce alcohol followed by aerobic fermentation using *Acetobacteracetii vat Europeans* for another 4 weeks, which produced 6–8% of acetic acid at the end of the processes. Then, the sample was left to mature at room temperature for 4 weeks. The final product, the liquid pineapple vinegar,will have a pungent smell with a slightly brownish color. The sample was then kept at 4 °C for further use.

### In vitro cytotoxicity study

For the in vitro study, it is necessary to freeze dry the sample. The pineapple vinegar prepared in previous step was extracted using ethyl acetate (319902, Sigma Aldrich, USA) following the protocols described by Nishidai (2000) with slight modifications [19]. Briefly, 1.5 L of pineapple vinegar were gently mixed with ethyl acetate at room temperature at a ratio of 1:1 (v:v). The mixture was incubated for 5 min to allow the phases to separate. The ethyl acetate fraction (top layer) was separated from the immiscible layer using separatory funnel. The fraction was then evaporated using rotary evaporator (Büchi Rotavapor R-215, Switzerland). The extracted pineapple vinegar was then dissolved with cell culture media at a desired concentration.

### Cell culture

Mouse mammary gland cells, 4 T1 (CRL-2539, ATCC, USA), human mammary gland cells MDA-MB-231 (HTB-26, ATCC, USA) and murine leukemia virus induced YAC-1 (TIB-160, ATCC, USA) were purchased from the ATCC collection and cultured in RPMI 1640 (R8758, Sigma Aldrich, USA) containing 10% fetal bovine serum (FBS) (26140, Gibco, USA). The cells were grown at 37 °C in a humidified incubator with 5% CO_2._

### 3-(4,5-Dimethylthiazol-2-yl)-2,5-diphenyltetrazolium bromide (MTT) assay

The cytotoxicity of pineapple vinegar was measured with the MTT assay. Briefly, 4 T1 and MDA-MB-231 cells (of 8.0 × 10^4^cells/well) were seeded on a 96-well plate. Twenty-four hours after initial seeding, a two-fold serial dilution of seven different concentrations (700.00, 350.00, 175.00, 87.50, 43.75, 21.88, 10.94 mg/mL) of pineapple vinegar was added into the plate. After 48 h of treatment, the cell viability was measured by adding 20 μL of MTT solution (5 mg/mL) (475989, Merck, USA) in each well. After 3 h of incubation with the MTT solution, the solution was discarded and 100 μL of DMSO (472301, Sigma Aldrich, USA) was added into the plate in order to solubilize the MTT crystals. The reading was taken after 30 min at the wavelength of 570 nm using enzyme-linked immunosorbent assay (ELISA) plate reader (Bio-tek Instruments, USA). The assay was done in triplicates. The cytotoxicity result was analyzed using the formula given below:

$$ \left[\mathrm{Percentage}\ \mathrm{of}\ \mathrm{Cell}\ \mathrm{Viability}=\right[\mathrm{OD}\ \mathrm{Sample}/\mathrm{OD}\ \mathrm{control}\left]\times 100\%\right] $$

From the MTT result, two inhibitory concentration (IC) values after 48 h of treatment for 4 T1 cells have been selected to be used in the following assays.

### Cell cycle analysis

4 T1 cells were cultured at the concentration of 2.3 × 10^5^ cells/well and were treated with pineapple vinegar at two different concentrations, namely; 0.25 mg/ml and 0.32 mg/mL for 48 h. After that, they were trypsinized before centrifuged at 2000 rpm for 5 min and the pellet was collected and fixed with 70% ethanol for at least a week. On the respective day, the pellets were washed with 500 μL of phosphate buffer saline (PBS) and treated with 10 μg/mL of RNAse and Triton-X 100 (X100, Sigma Aldrich, USA) before stained with 10 μg/mL of propidium iodide (PI) (P4170, Sigma Aldrich, USA). After 15 min of incubation, the cells were analyzed using a fluorescence-activated cell sorter (FACS) flow cytometry (Becton Dickinson, USA).

### Annexin V analysis

The apoptotic state of the cells was determined using fluorescein isothiocyanate (FITC) Annexin-V Apoptosis detection kit (556547, BD Pharmingen, USA) and the assay was done according to the user guideline provided. In brief, 4 T1 cells (2.3 × 10^5^ cells/well) were treated with pineapple vinegar at the concentrations of 0.25 mg/mL and 0.32 mg/mL for 48 h. Then, the cells were double-stained with PI and FITC for 15 min in the dark and analyzed using a FACS-Calibur flow cytometer (Beckman Coulter, USA). The experiment was carried out in triplicates.

### In vitro scratch assay

The assay was performed using the wound healing method as described in previous study [20]. In brief, 4 T1 cells were seeded on a 6 well plate and left overnight to full confluency. On the next day, with a sterile yellow tip, a linear scratch was introduced in the middle of the wells. Then, the old media was replaced with the new media containing different concentrations of pineapple vinegar. The closure rate of the scratch was observed and captured every few hours up to 24 h using a light microscope (Nikon, Japan). The rate of wound closure was calculated using the following formula:

$$ \mathrm{Percentage}\ \mathrm{of}\ \mathrm{scratch}\ \mathrm{closure}=\frac{\left(\mathrm{Area}\ \mathrm{of}\ \mathrm{wound}\ \mathrm{at}\ 0\ \mathrm{hour}-\mathrm{area}\ \mathrm{wound}\ \mathrm{at}\kern0.5em n\kern0.5em \mathrm{hour}\right)}{\left(\mathrm{Area}\ \mathrm{of}\ \mathrm{wound}\ \mathrm{at}\ 0\ \mathrm{hour}\right)}\mathrm{x}\ 100\% $$

### The chemo-preventive experiment design

The chemo-preventive effect of pineapple vinegar was evaluated using 4–6 weeks old BALB/c female mice with average weight of 20–22 g. This study was approved by the Institutional Animal Care and Use Committees (IACUC) of UPM (UPM/IACUC/AUP-R097/2014). All animal procedures were performed strictly according to the protocol by the IACUC of UPM. The mice were randomly divided into 4 groups with 8 mice per group. The mice were acclimatized for one week (22 ± 1 °C; 12-h dark/light cycle) and they were given distilled water and standard pellet diet ad libitum*.* Then, the mice were separated into groups (below) and pre-treated with either distilled water or pineapple vinegar for 6 weeks and post-treated for 4 weeks via oral gavage based on the course of time conducted during the pilot study. Two ml/kg BW was chosen as the highest concentration as it is the common maximum concentration used by all in vivo vinegar studies done previously while the 0.08 ml/kg BW was calculated based on the common concentration of vinegar consumed by human (1 tablespoon of vinegar diluted in 1 glass of water).

- Untreated (UT): Induced mice, given distilled water throughout the study (untreated);
- Pineapple vinegar low concentration (PL): Induced mice, pretreated with pineapple vinegar (0.08 ml/kg BW);
- Pineapple vinegar high concentration (PH): Induced mice, pretreated with pineapple vinegar (2 ml/kg BW).

At the end of 6th week, all mice were inoculated with the 4 T1 cells via subcutaneous (s.c) injection of 1 × 10^5^ cells in 100 μL PBS. The treatment continued for another 4 weeks. At the end of the experiment, all mice wereanesthetized with 2% isoflurane (1349014, Merck, USA) and sacrificed by cervical dislocation. Tumor, spleen and blood were collected from the mice prior to the following assays.

### Body weight and tumor weight analysis

The body weights of the mice were measured once a week until the end of the study. Tumors were weighed and washed in PBS before they were chopped into smaller pieces and kept in liquid nitrogen for further use.

### Immunophenotyping by flow cytometry

Briefly, the harvested spleens were washed and meshed through 70 mm cell strainers (SPL, Korea) in PBS. The supernatants were centrifuged at 2000 rpm for 15 min before the pellets were lysed using ammonium chloride (NH_4_Cl) buffer. Then, the splenocytes were stained with four different antibodies; CD3 (AB24948, Abcam, UK), CD4 (AB86859, Abcam, UK), CD8 (AB39850, Abcam, UK), NK1.1 (AB25352, Abcam, UK) and macrophage (AB105155, Abcam, USA) before they were subjected to flow cytometry analysis using a FACS Calibur flow cytometer (BD, USA).

### Terminal Deoxynucleotidyl transferase dUTP Nick end labeling (TUNEL) assay

The TUNEL assay was carried out using the DeadEnd™ colorimetric TUNEL System (G7360, Promega, USA) according to the manufacturer’s protocol. In brief, tumors were excised and embedded on slide. Then, the slides were deparaffinized in xylene twice for 5 min before they were rehydrated in decreasing concentrations of ethanol (100, 95, 85, 70 and 50%) beforethey were washed in PBS. To detect apoptotic cells, the slides were fixed in 4% paraformaldehyde and permeabilized using proteinase K before they were fixed again in 4% paraformaldehyde. Later, the slides were then equilibrated using the equilibration buffer and labeled using terminal deoxynucleotidyl transferase (TdT). Next, the slides were blocked in hydrogen peroxide before being incubated with streptavidin horseradish peroxidase (HRP). The slides were then developed using 3,3′-Diaminobenzidine (DAB) and mounted in glycerol and they were viewed under a bright-field inverted microscope (Nikon, Japan). The degree of DNA fragmentation was measured based on the presence of dark brown cells (apoptotic cell indicator) against a light brown background.

### Metastasis analysis using clonogenic assay and bone marrow test

The anti-metastatic potential of pineapple vinegar was investigated using clonogenic assay and bone marrow test. Briefly, the mice were sacrificed before their liver, spleen, lung and leg were removed under sterile condition, washed with PBS and were prepared according to the assays respectively. The clonogenic assay was done according to the previous study [19]. In brief, the liver, spleen and kidney were chopped into smaller pieces (<1cm^3^). Then, each organ was incubated in PBS and collagenase D for 20–30 min at 37 °C and the mixtures were mixed thoroughly every 5 min. Next, the solution was passed through 70 mm cell strainer (SPL, Korea) and spun at 2000 rpm for 10 min. The pellet was then washed with PBS and re-suspended in 10 ml suspension medium (RPMI 1640, 10% FBS, 1% penicillin-streptomycin, 60 μM 6-thioguanine). Six serial dilutions were made in 6-well plates and the plates were incubated for 10 days. On the 10th day, the plates were fixed with methanol and stained with crystal violet. The results were obtained by counting the colony by naked eyes.

The bone marrow-derived cell (BMDC) - cancer cell metastasis assay was done according to a previous study with slight modifications [21]. Briefly, the femurs were harvested and washed in PBS. Then, 100 μL of PBS was used to flush out the bone marrow of femur and a smear was done by sliding the liquid onto the glass slides. The slides were air dried at room temperature for half an hour before they were fixed in methanol for another half an hour. Next, the fixed slides were stained with undiluted Giemsa solution (48900, Sigma Aldrich, USA) for 15 min before they were washed with distilled water for few times and leave to air dry overnight. The slides were viewed at 100x magnification under a light microscope (Nikon, Japan).

### Lactate dehydrogenase (LDH) Splenocyte cytotoxicity assay

This assay was done according to the previous study [20]. The spleens from all treatment groups were harvested before they were meshed and incubated with YAC-1 cells overnight. CytoTox 96 nonradioactive cytotoxicity assay kit (G1780, Promega, USA) was used to determine the cytotoxicity activities. The splenocytes were seeded at the ratio of splenocytes (effector spontaneous) to YAC-1 cells (target spontaneous) being 2 to 1 and 5 to 1. The cells were incubated for 24 h in a 90% humidified 37 °C incubator supplemented with 5% CO_2_. After 24 h of incubation, a lysis solution was added to the wells. After 45 min, the media was removed and 50 μL of the reconstituted substrate mix was added and it was incubated for 30 min at room temperature. After 30 min, 50 μL of stop solution was added and the absorbance was taken at 490 nm using a μquant microplate reader (Bio-Tek Instruments, USA). The percentage of cytotoxicity was calculated using the equation given in the manual protocol.

$$ \%\mathrm{Cytotoxicity}=\frac{\mathrm{Experimental}-\mathrm{Effector}\ \mathrm{Spontaneous}-\mathrm{Target}\ \mathrm{Spontaneous}}{\mathrm{Target}\ \mathrm{Maximum}-\mathrm{Target}\ \mathrm{Spontaneous}}\times 100 $$

### Real time polymerase chain reaction (RT-PCR) analysis

Equal amounts of total ribonucleic acid (RNA) of tumor samples were extracted using RNeasy mini kit (74104, Qiagen,Germany) and reverse transcribed to complementary DNA (cDNA) using iScript™ cDNA Synthesis Kit (1708890, Bio-Rad,USA). The cDNA was then subjected to the RT-PCR analysis using iQ5 RT-PCR machine (Bio-Rad, USA) and the results were analyzed using iQ5 Optical System Software. The primer sequences used in the study were given in Additional file 1: Table S1.

### Cytokine assay

The blood was collected from each mouse using BD Microtainer® Tubes (Becton Dickinson, USA) and spun at 14000 rpm for 15 min and kept in -20 °C until further use. On the respective day, the serums were diluted 10 times using assay diluent buffer and they are subjected to the following ELISA cytokine assays; interleukin-2 (IL-2) (DY402, R&D Systems, USA), interleukin-1 beta (IL-1β)(DY401, R&D Systems, USA), interleukin-10 (IL-10) (DY417, R&D Systems, USA) and interferon gamma (IFN-γ) (DY485, R&D Systems, USA). All assays were done according to the R&D Systems Mouse Cytokine kit manual protocols (R&D Systems, USA).

### Protein preparation from tumor

The tumors were harvested, snapped freeze and kept in -80 °C until use. On the respective day, the tumors were weighed and meshed using a mortar and pestle with liquid nitrogen. Then, the tumors were lysed in radioimmunoprecipitation assay (RIPA) buffer (150 mM sodium chloride, 1.0% NP-40 or Triton X-100 0.5% sodium deoxycholate, 0.1% sodium dodecyl sulfate (SDS) and 50 mM Tris, pH 8.0 mixed with protease inhibitor cocktail) (Pierce, Thermo Fisher Scientific, USA). The protein concentrations were measured using Bradford reagent (Bio-Rad, USA). Then, the protein samples were kept in -80 °C for further use.

### Western blot analysis

By using SDS page, equal amounts of protein were separated and transferred to nitrocellulose membrane (PALL, USA). The membrane was then blocked with 5% non-fat milk (NB0669, Biobasic, Canada) overnight. The next day, the membrane was washed with tris-buffered saline (TBS) and Polysorbate 20 (TBST) (10 mM Tris, 140 mMNaCl, 0.1% Tween-20, pH 7.6) and incubated in primary antibody for 1 h at 4 °C followed by washing with TBST before it was incubated with appropriate secondary antibody for another hour. Then, it was washed again and incubated with HRP substrate for 10 min before it was viewed using a chemiluminescence imager (UVP, USA). Beta actin (β-actin) (AB8226, Abcam, USA) was used as a housekeeping control. The results obtained were analyzed using Vision Work LS Analysis software (UVP, USA).

### Proteome profiler assay (angiogenesis)

The effect of pineapple vinegar on the angiogenic process of 4 T1 cancer cells was investigated using proteome profiler kit (ARY015, R&D System, USA). Briefly, the membranes were blocked with blocking buffer while the protein samples were incubated with the detection antibody at room temperature for an hour. Next, the protein mixtures were transferred to the membrane and incubated at 4 °C overnight. On the next day, the membranes were washed three times using washing buffer and incubated with streptavidin-HRP for 30 min. Lastly, the membranes were washed again 3 times before the substrate was added to develop chemiluminescence. The membranes were then viewed using Chemi Doc XRS (Bio-Rad).

### Statistical analysis

All experiments were carried out in three biological replicate (*n* = 3). Quantitative data were expressed as mean ± SD and were analyzed using one-way ANOVA, SPSS16. *P* values of < 0.05 were considered statistically significant.

## Results

### Pineapple vinegar inhibited the viability of 4 T1 cells in vitro

Colorimetric tetrazolium reduction test (MTT) was carried out to study the cytotoxicity of pineapple vinegar against the 4 T1 and MDA-MB-231 breast cancer cell lines. The cytotoxicity effect was determined from the 50% inhibitory concentration (IC_50_) and 75% inhibitory concentration (IC_75_) at 48 h treatment. Pineapple vinegar showed IC_50_ values of 0.25 ± 0.03 and 0.27 ± 0.02 mg/mL to4 T1 and MDA-MB-231 triple negative cell lines at 48 h. In addition, the IC_75_ values were 0.32 ± 0.02 and 0.35 ± 0.03 mg/mL to 4 T1 and MDA-MB-231 cells, respectively. Overall, the IC_50_ and IC_75_ value of pineapple vinegar on murine and human triple negative mammary gland cancer cells are similar and not significantly different (*p* > 0.05).

### Apoptosis induction analysis of pineapple vinegar treated 4 T1 cells

**Fig. 1:**
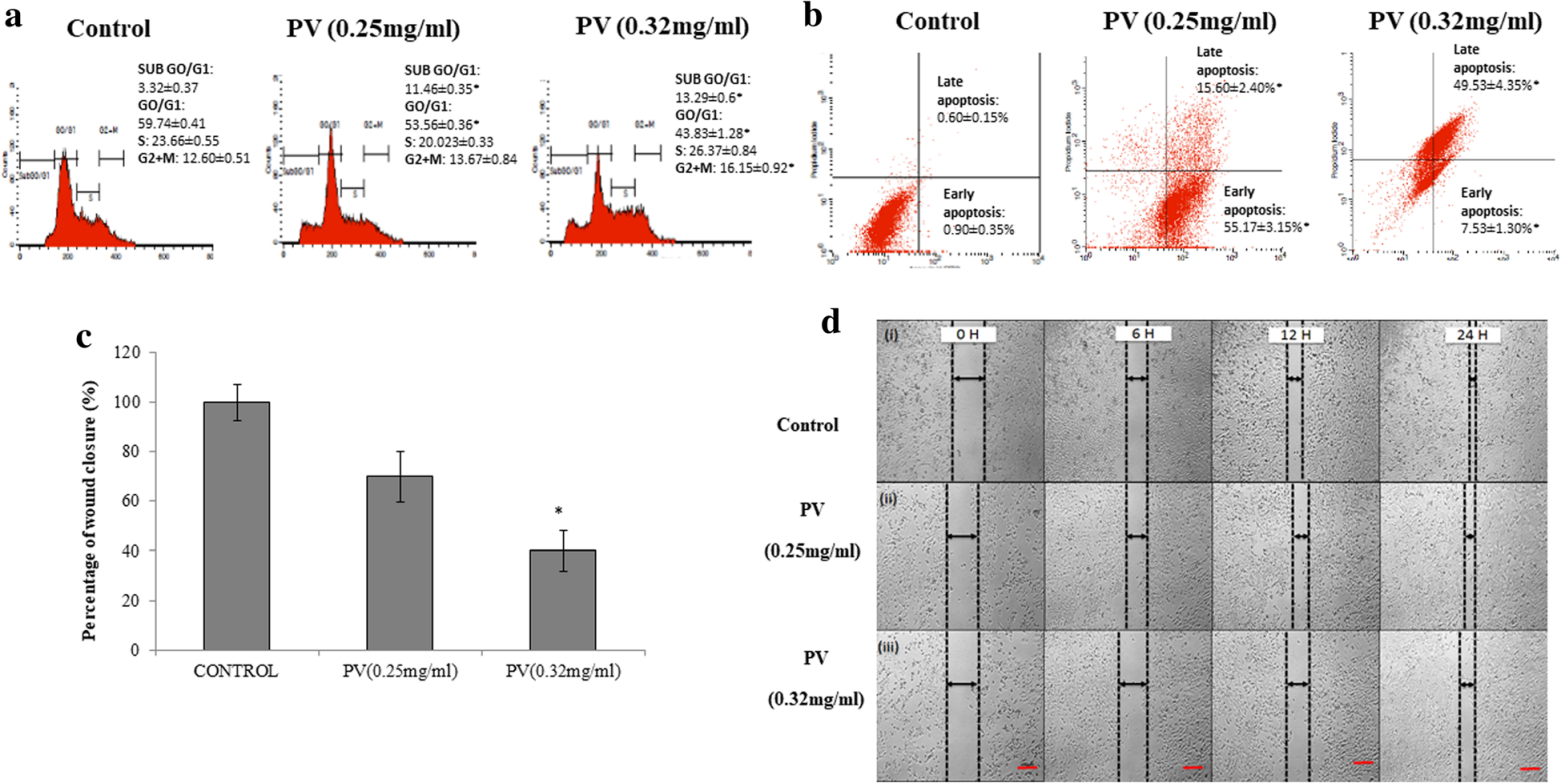
**a** Cell cycle progression and **b** AnnexinV/PI apoptosis detection of untreated and pineapple vinegar treated 4 T1 cells at 48 h. In vitro scratch assay**. c** The size of the wound after 24 h of incubation. **d** Images of wounds at 0, 6, 12, and 24 h show characteristic protrusion of pineapple vinegar at different treatment, (i) treated with PBS (control), (ii) treated with pineapple vinegar (0.25 mg/mL) and (iii) treated with pineapple vinegar (0.32 mg/mL)(magnification: × 40, red line indicates 100 μm). Note: The data presented were representative of biological replicates obtaining from three independent experiments ± SD (*n* = 3). Significant values were calculated against control group (*P < 0.05)

The cell cycle regulation by pineapple vinegar was investigated using a cell cycle analysis. Figure 1a showed the normal distribution in the untreated 4 T1 cells (control) at 48 h treatment. As the concentration of the pineapple vinegar increased to 0.32 mg/mL, the proportion of S phase increased to 26.37 ± 0.84% as compared to the control (23.66 ± 0.55%). Sub G0/G1 phase marked a significant increase for treatment with pineapple vinegar (IC_50_ at 11.46 ± 0.35%; IC_75_ at 13.46 ± 0.60%) as compared to the control (3.32 ± 0.37%). These results suggest that the treatment with pineapple vinegar was able to inhibit the growth of 4 T1 cells through apoptosis by the significant increment in the percentage of cells in hypo diploid subG0/G1 population (2nDNA).

To further support the induction of apoptosis by pineapple vinegar, Annexin V assay was done and the results were displayed in Fig. 1b. In quadrant I (FITC negative, PI negative), a high population of viable cells was observed in the untreated control group (98.40 ± 0.22%). A significant increase in the early apoptosis cell population was observed in the quadrant II (FITC positive, PI negative) in the pineapple treated groups (0.25 mg/mL PV = 55.17 ± 0.24%; 0.32 mg/mL PV = 7.53 ± 0.29%). The treatment with 0.32 mg/mL of pineapple vinegar showed a drastic increment (49.54 ± 0.95%) in the cell population in quadrant III (FITC negative, PI positive) and IV (FITC positive, PI positive) as compared to the 0.25 mg/mL of PV, which only induced 15.60 ± 0.16% of 4 T1 cells under late apoptosis stage. These results imply that pineapple vinegar induced apoptosis in a dose dependent manner and as such, pineapple vinegar is a potent inducer of apoptosis and necrosis and could trigger events leading to apoptotic cell death.

### Pineapple vinegar inhibited the migration of 4 T1 cells in vitro

The effect of pineapple vinegar in inhibiting the migration of 4 T1 cells was investigated through in vitro scratch assay. After 24 h of incubation, the rate of wound closure decreased as the dose increased (Fig. 1c and d). The pineapple vinegar was able to inhibit the closure of the scratch of 4 T1 cells to about 30% at the concentration of 0.25 mg/mL and around 60% at the concentration of 0.32 mg/mL at a time dependent manner.

### Pineapple vinegar inhibited the growth of tumor in vivo

**Fig. 2:**
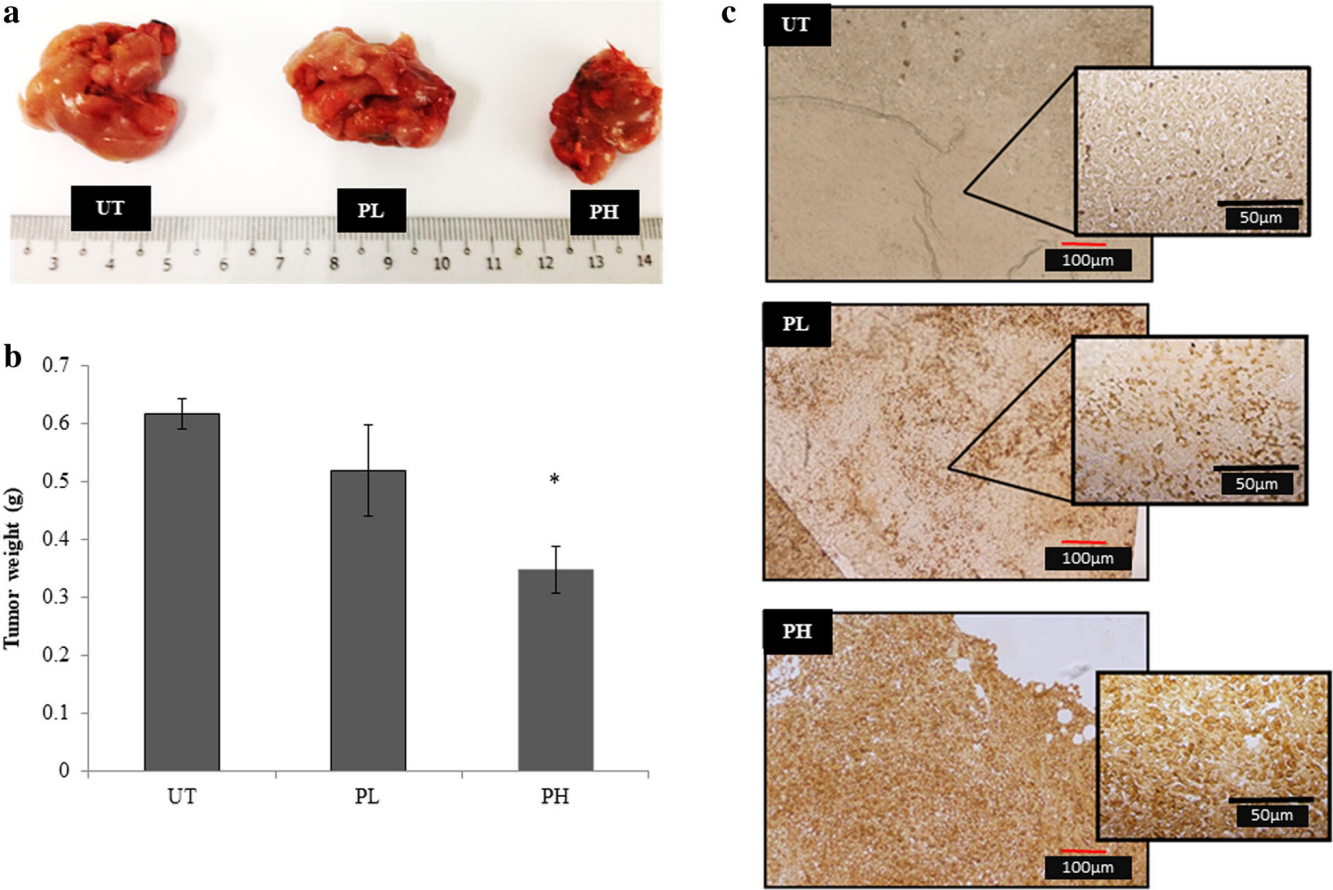
**a** Representative tumor image and **b** tumor weightof 4 T1 cells of theuntreated (UT), 0.08 mL/kg BW pineapple treated mice (PL) and 2 mL/kg BW pineapple treated mice (PH) groupsafter 28 days of treatment. **c** BrdU labelledDNA fragmentation in the tumor ofthe untreated (UT), 0.08 mL/kg BW pineapple treated mice (PL) and 2 mL/kg BW pineapple treated mice (PH) groups detected by Tunel assay (for large image, magnification: × 40, red line indicates 100 μm; for zoom in image, magnification × 100, black line indicates 50 μm). Note: The data presented were representative of biological replicates obtaining from of three independent experiments ± SD (n = 3). Significant values were calculated against UT group (*P < 0.05)

The anti-tumor activity of pineapple vinegar in mice was assessed using tumor weight data (Fig. 2a and b). Figure 2b showed that the tumor weight was slightly lower (15.88%) in PL group and significantly lower (43.60%) in PH group as compared to the UT group.

### Pineapple vinegar induced apoptosis in tumor

TUNEL assay was performed to study the apoptotic effect of pineapple vinegar in the tumor. Figure 2c showed that only TUNEL positive brown spots (DNA fragmentation) were observed in the UT group and the presence of TUNEL positive dark brown spots increased in the treatment groups as the concentration of the samples increased. The frequency of apoptotic events in mice was higher in PH group as compared to UT and PL groups (Fig. 2c).

### Pineapple vinegar enhanced the immunity in vivo

**Fig. 3:**
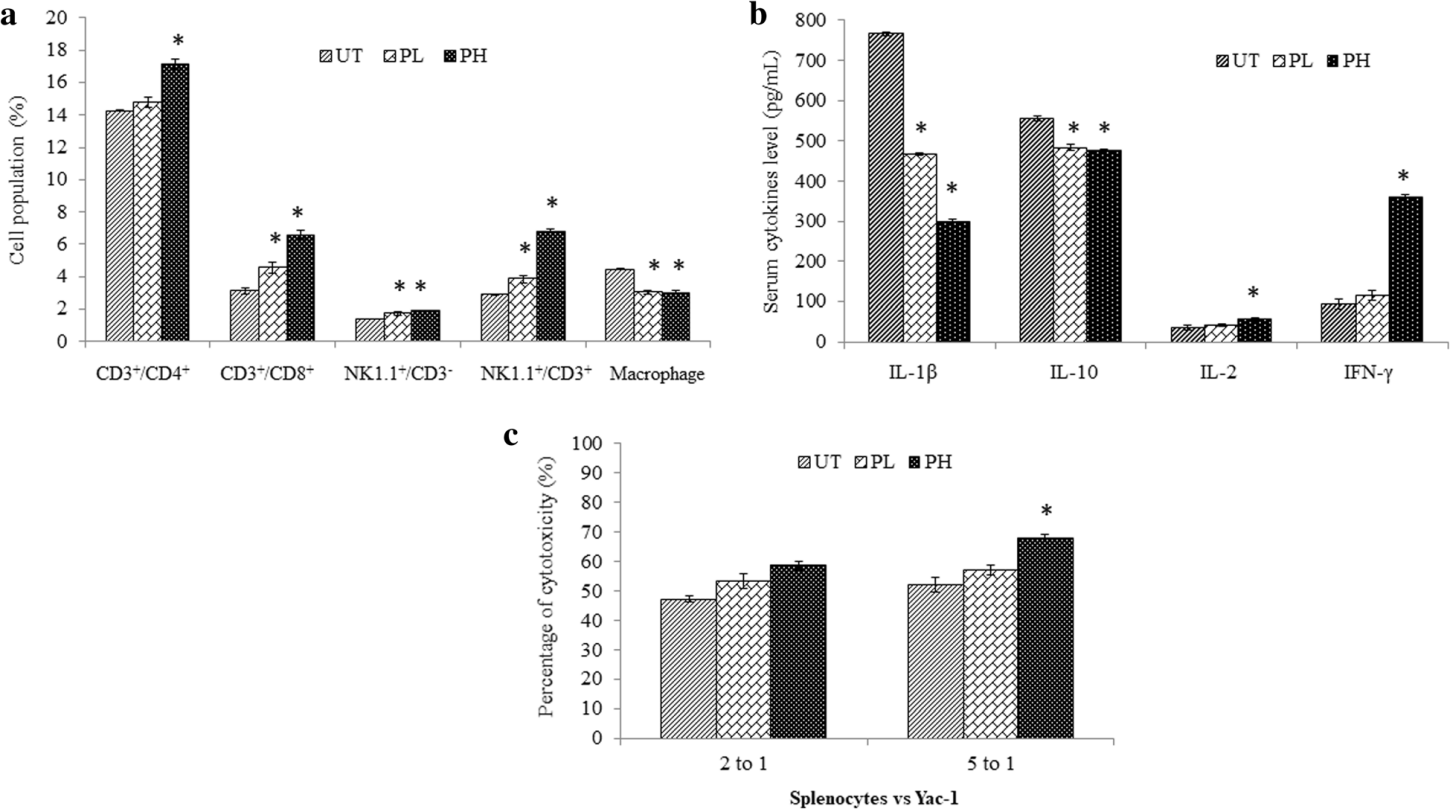
**a** Flow cytometry immunophenotyping of CD3^+^/CD4^+^, CD3^+^/CD8^+^, NK1.1^+^/CD3^−^,NK1.1^+^/CD3^+^ and macrophage population of splenocyte; **b** serum IL-1β, IL-10, IL-2 and IFN-γ cytokines level; **c** cytotoxicity of splenocytes against Yac-1 cells at ratio of 2 to 1 and 5 to 1 foruntreated (UT), 0.08 mL/kg BW pineapple treated mice (PL) and 2 mL/kg BW pineapple treated mice (PH) after 28 days of treatment. Note: The data presented were representative of biological replicates obtaining from three independent experiments ± SD (n = 3). Significant values were calculated against UT group (*P < 0.05)

As depicted in Fig. 3a, pineapple vinegar reduced the concentration of pro-inflammatory cytokines, IL-1β from 766.67 ± 5.20 pg/mL (UT) to 466.67 ± 3.20 pg/mL in PL group and 300.00 ± 4.60 pg/mL in PH. Similar trend was observed in the IL-10 level where a significant decrease was observed in the pineapple vinegar groups as compared to the UT group (UT = 555.56 ± 3.40 pg/mL; PL = 483.33 ± 7.20 pg/mL; PH = 477.54 ± 5.40 pg/mL). Conversely, significant increase of IL-2 andIFN-γ levels were observed in PH group where IL-2 level of PH increased from 35.09 ± 5.94 pg/mL in the UT group to 56.14 ± 5.21 pg/mL and from 94.21 ± 11.91 in the UT group to 361.40 ± 4.56 pg/mL for IFN-γ level.

Immunophenotyping was done to further investigate the immunostimulatory effect of pineapple vinegar. As summarized in Fig. 3b, pineapple vinegar increased the level of CD4^+^/CD3^+^, CD8^+^/CD3^+^ and both natural killer cell populations (NK1.1^+^/CD3^+^ and NK1.1^+^/CD3^−^) and decreased the level of macrophage in the mouse splenocytes significantly. It was observed that the level of CD4^+^/CD3^+^ was significantly increased in PH group from 14.25 ± 0.04% (UT) to 17.14 ± 0.29% (PH). A marked increment in the level of CD4^+^/CD3^+^ (UT = 3.12 ± 0.20%; PL = 4.58 ± 0.34%; PH = 6.59 ± 0.28%), NK1.1^+^/CD3^−^(UT = 1.39 ± 0.04%; PL = 1.74 ± 0.14%; PH = 1.91 ± 0.01%), and NK1.1^+^/CD3^+^ (UT = 2.89 ± 0.06%; PL = 3.92 ± 0.31%; PH = 6.82 ± 0.09%) were demonstrated in both pineapple vinegar treatment groups as compared to the UT group. On the other hand, the level of macrophage was found to drop significantly when treated with pineapple vinegar (UT = 4.45 ± 0.050%; PL = 3.03 ± 0.10%; PH = 3.01 ± 0.10%).

These results were further verified using a standard LDH cytotoxicity assay to quantify the cytotoxicity of the pineapple vinegar-treated and non-treated splenocytes against YAC-1 cells. Figure 3c showed that the exposure of splenocytes with Yac-1 cells at the ratio of 2 to 1 raised the cytotoxicity significantly to 53.34 and 58.70% for PL and PH groups respectively. The cytotoxicity was further increased when the number of splenocytes increased from 2 times to 5 times against the total cell number of YAC-1 cell.

### Pineapple vinegar regulated several inflammatory markers in vivo

**Fig. 4:**
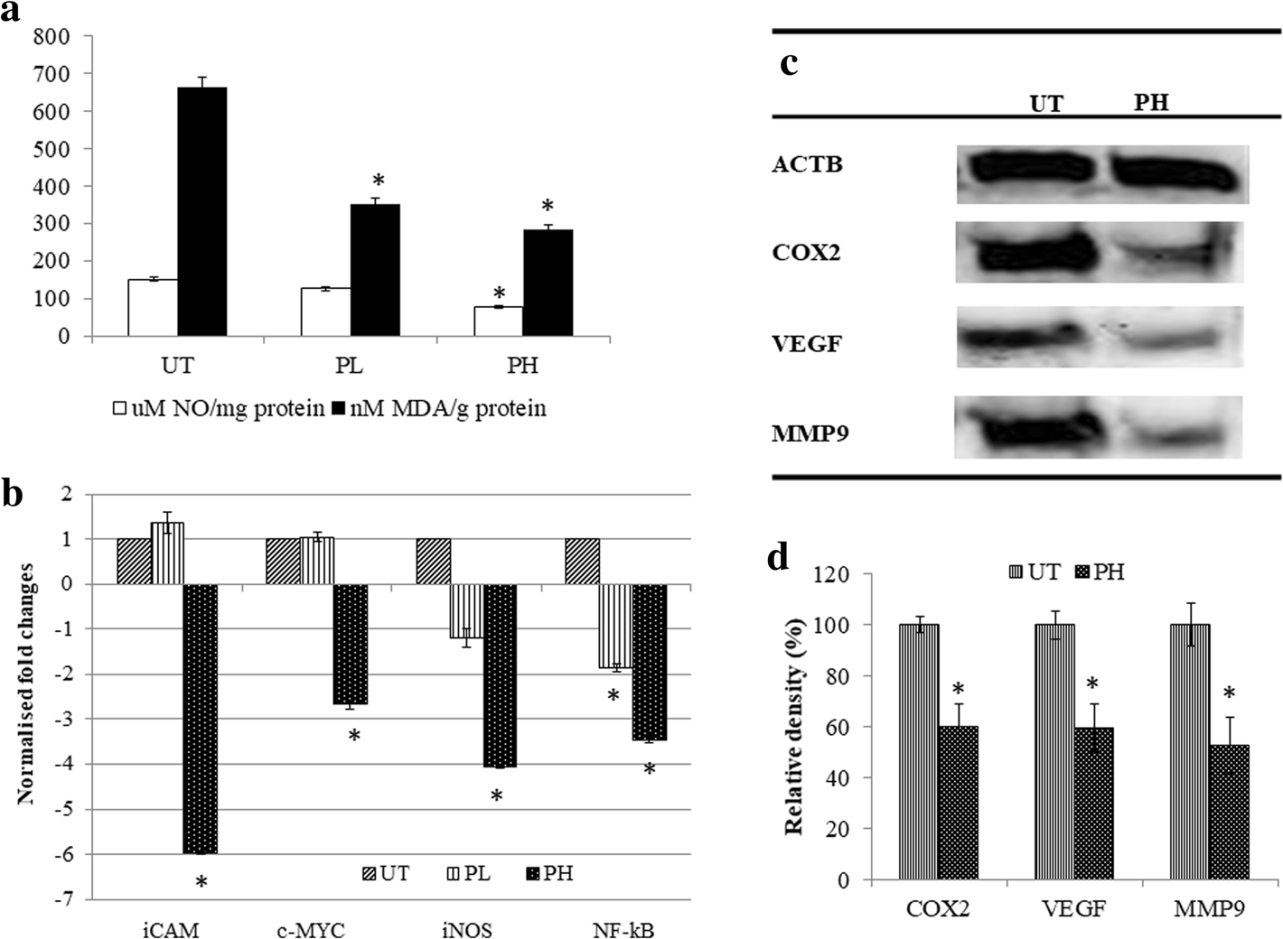
**a** Tumor NO and MDA levels; **b** qPCR analyses on iCAM, c-MYC, iNOS and NF-kB genes in the tumor; **c** representative blot and **d** relative density for western blot analysis of COX2, VEGF and MMP9 protein expressions in the tumor of the untreated (UT) and 2 mL/kg BW pineapple vinegar (PH) in mice induced with 4 T1 breast cancer cells. β-actin was used as reference protein. Note: The data presented were representative of biological replicates obtaining from three independent experiments ± SD (n = 3). Significant values were calculated against UT group (*P < 0.05)

The ability of pineapple vinegar in reducing the inflammation in the tumors was investigated through the ability of the samples to decrease the level of nitric oxide (NO) and lipid peroxidation in tumor homogenate samples. As depicted in Fig. 4a, pineapple vinegar treated groups were able to reduce the level of NO (UT = 149.73 ± 5.39; PL = 125.74 ± 4.18; PH = 78.47 ± 3.36 μM NO/mg protein) and malonaldehyde (MDA) (UT = 661.78 ± 28.79; PL = 352.32 ± 16.01; PH = 281.86 ± 13.42 nM MDA/g protein) significantly. The result was further investigated through the regulation of several inflammatory-related genes and they were demonstrated in Fig. 4b-d. Results showed that the treatment with pineapple vinegar was able to reduce the expressions of inducible nitric oxide synthases (iNOS) (PL = 1.2 fold; PH = 4 fold) and nuclear factor kappa-light-chain-enhancer of activated B cells (NF-κβ) (PL = 1.9 fold; PH = 3.5 fold) genes as compared to the UT group (Fig. 4b) and inhibited the cyclooxygenase-2 (COX-2) protein by 2 fold in PH group (Fig. 4c and d).

### Pineapple vinegar inhibited the metastasis process in vivo

**Fig. 5:**
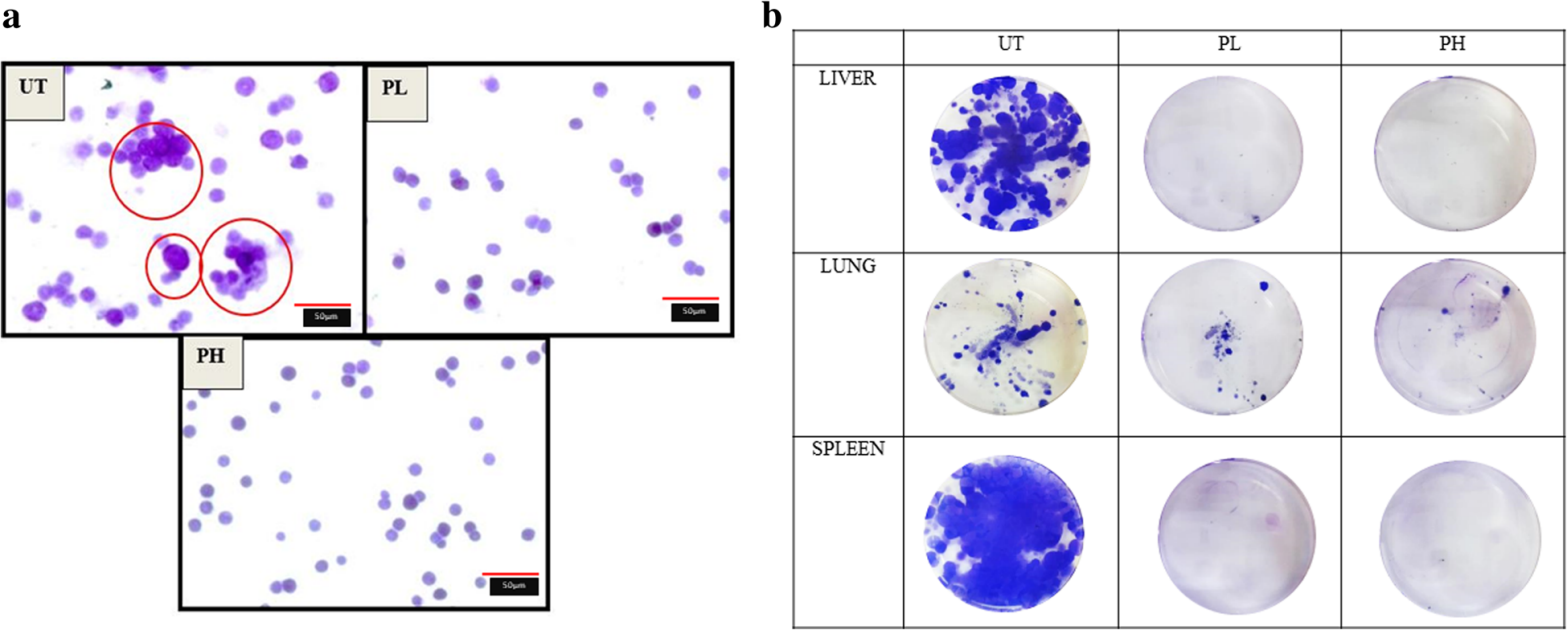
Metastasis of 4 T1 cells to **a** bone marrow (magnification: × 100, red line indicates 50 μm). Note: The data presented were representative of biological replicates obtaining from three independent experiments ± SD (n = 3), **b** lung, liver and spleen of untreated (UT), 0.08 mL/kg BW pineapple treated mice (PL) and 2 mL/kg BW pineapple treated mice (PH). Metastasis of 4 T1 cells to organs was measured by clonogenic assay. Circle = metastatic cells in bone marrow smear

To further elucidate the anti-metastasis effect of the pineapple vinegar, the bone marrow smearing assay and the clonogenic assay have been carried out. Figure 5a showed the invasion of the cancer cells into the bone marrow in the UT group while no abnormal cells was observed in the bone marrow of the treated groups. A similar pattern was observed in the clonogenic assay as depicted in Fig. 5b where only few colonies formed in the lungs of pineapple vinegar treatment group and no colonies were spotted in the other two organs (liver and spleen).

**Fig. 6:**
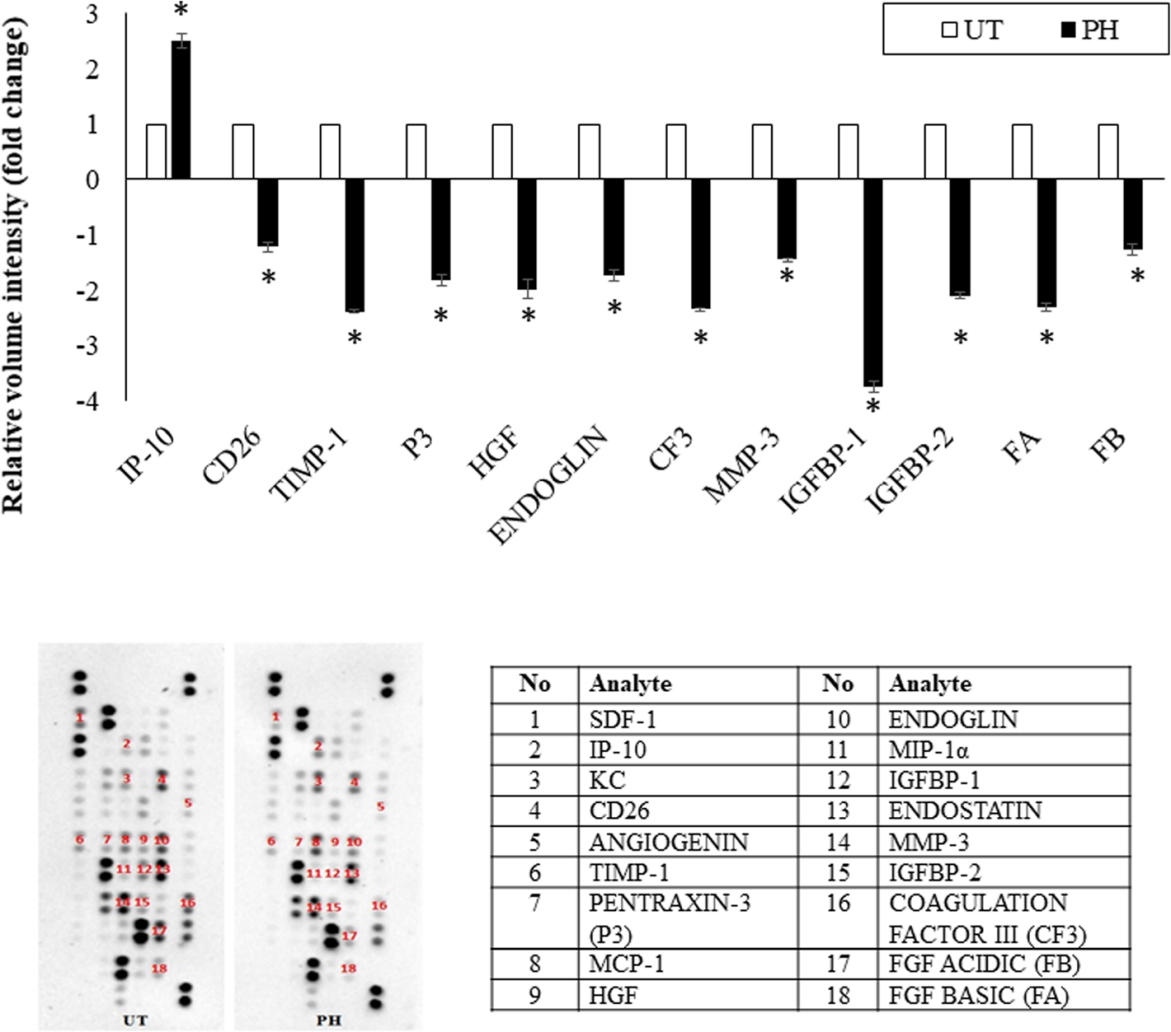
Angiogenesis proteome assay of tumor in the untreated (UT) and 2 ml/kg BW pineapple treated mice (PH) groups. Note: The data presented were representative of biological replicates obtaining from three independent experiments ± SD (n = 3). Significant values were calculated against UT group (*P < 0.05)

RT-PCR, western blot and proteome profiler were done to understand the effect of pineapple vinegar on the expression of genes involved in metastasis. Figure 4b-d demonstrated the significant down-regulation of c-Myc (2.7 fold), intercellular adhesion molecule 1(ICAM-1) (5.9 fold), vascular endothelial growth factor (VEGF) (1.6 fold), and matrix metalloproteinases 9 (MMP9) (1.9 fold). Moreover, the regulation of several angiogenesis-related proteins is depicted in Fig. 6. From 18 angiogenesis proteins found to be regulated by pineapple vinegar, only 12 were significantly different. From the results, PH group showed a remarkable increase in interferon γ-induced protein 10 kDa (IP-10) protein (IP-10 = 1.5 fold) and a significant decreased in several angiogenesis protein (cluster of differentiation 26 (CD26) = 2.2 fold; tissue inhibitors of metalloproteinases 1(TIMP1) = 3.4 fold; P3 = 2.8; hepatocyte growth factor (HGF) = 2.9; ENDOGLIN = 2.7; CF3 = 3.3; matrix metalloproteinases 3 (MMP-3) = 2.5; insulin-like growth factor-binding protein 1 (IGFBP-1) = 4.8; insulin-like growth factor-binding protein 2 (IGFBP-2) = 3.1; FA = 3.3; FB = 2.3).

## Discussion

The chemoprevention via non-toxic agents could be a promising approach to prevent the incidence of cancer. Many naturally occuring agents have shown chemopreventive potential in a variety of bioassay systems and animal models [20]. Vinegar, on the other hand, has been utilized since ancient time as a cooking ingredient and folk medicine to treat many diseases such as cardiovascular disease and cancer [13–15]. Based on the in vitro and in vivo results presented in this study, it showed that pineapple vinegar possessed cytotoxic effect against 4 T1 cells and the pre-treatment with pineapple vinegar was able to reduce the tumor size significantly. These effects might be contributed through its ability to induce apoptosis, reduce inflammation and inhibit metastasis. Apoptosis is a programmed cell death that plays a significant role in cancer treatment [22]. The effect of pineapple vinegar to reduce the viability of 4 T1 breast cancer cells has been proven through the MTT screening result. The potential of pineapple vinegar to induce apoptosis was then verified through the cell cycle analysis and by detecting the externalization of phosphatidylserine using Annexin V analysis. In addition, in vivo TUNEL assay revealed the capability of pineapple vinegar to induce DNA fragmentation, animportant indicator of apoptosis execution process [22].

Immune suppression occurs frequently in cancer patient as the tumor cells normally inhibit the immune surveillance and reduce recognition by the immune cells [23]. T cells control the tumor progression by secreting pro-apoptotic cytokines [23]. Among those cytokines, IL-2 and IFN-γ are known as anti-tumor cytokines, which help to execute cancer cells and activate T and NK cells cytotoxicity [24, 25]. The activation of IL-2 leads to the induction of IFN-γ and the regulation of CD4+/CD3+ and CD8+/CD3 + cells by the activation of T helper cell [24]. The activation of these T helper, CD4+/CD3+, and CD8+/CD3 + cells may help to inhibit or delay the tumor progression. Based on the result, the increase of CD4+/CD3+, CD8+/CD3+ and NK cells in pineapple vinegar treatment groups were consistent with the increase of IL-2 and IFN-γ. This finding showed that the anti-tumor activity of pineapple vinegar may be contributed by the activation of cell mediated immunity by pineapple vinegar.

Apart from the suppression of anti-tumor immunity, inflammation is always connected to the promotion of cancer cells proliferation. Therefore, the inhibition of the inflammation process is favorable in cancer treatment [26]. In this study, the effect of pineapple vinegar in reducing tumor inflammation has been investigated. During the tumor development, a pro-inflammatory microenvironment that favors the survival of cancer cell form around the tumors [26]. This condition primes the body immune system to produce pro-inflammatory cytokines such as IL-1 beta (IL-1β) and subsequently activates NF-κβ [27]. The activation of NF-κβ promotes the up-regulation of COX-2 and iNOS, which subsequently promote the production of NO [27]. As the level of NO increases, the burden of the oxidative stress on the body is amplified and as a result, the body will release lipid peroxidative markers such as MDA. This severe inflammation was found to favor the progression of cancer as the inflammatory microenvironment serves to further potentiate tumor growth [26]. Based on the results, it was observed that the down-regulation of NF-κβ occurred concurrently with IL-1β and IL-10 depletion in the treated groups. The significant down-regulation of NF-κβ also brought about the significant down-regulation in COX-2 and iNOS levels and the decrement in the production of NO and MDA in the treated groups as well.

4 T1 cell is an aggressive and highly metastatic murine breast cancer cell line [28]. 4 T1 cells were able to metastasize into the bone marrow, liver, spleen and lung of the UT mice as shown in the bone marrow smearing and clonogenic assays. Conversely, the treatment with pineapple vinegar demonstrated an anti-metastasis effect as the number of colonies detected in the liver, lung and spleen of the pineapple vinegar-treated mice were decreased or not detected. These results supported the in vitro anti-migration and anti-invasion abilities of pineapple vinegar against 4 T1 cells. To understand the anti-metastatic mechanism regulated by pineapple vinegar, the expression of c-Myc, ICAM-1, VEGF and MMP9, which are involved in the progression of cancer and highly expressed in cancer cells [29] were evaluated. A significant reduction in the expression of these genes was observed in both PH and PL groups. The inhibition of these genes may be resulted from the down-regulation of NF-κβ by pineapple vinegar as previous studies have shown a strong correlation between the regulation of the NF-κβ gene on several genes that involve in the metastasis processes including c-Myc, ICAM-1, VEGF and MMP9 [29].

Angiogenesis is one of the important steps in the metastasis process in breast cancer progression [30]. Based on the angiogenesis proteome profiler result, pineapple vinegar was able to significantly regulate several angiogenesis-related proteins such as IP10, CD26, TIMP-1, PENTRAXIN-3, HGF, ENDOGLIN, IGFBP-1 and -2, ENDOSTATIN, MMP-3, COAGULATION FACTOR III, fibroblast growth factor (FGF) acidic and basic. Previous study found that the activation of IFN-γ helped to activate IP-10, one of the anti-tumor cytokines involves in the production of T cell and the inhibition of FGF production [31]. FGF promotes the tumor growth through the growth of new blood vessel [32]. In this study, the up-regulation of IP-10 and the down-regulation of FGF basic and acidic in the tumor of pineapple vinegar treated mice were strongly associated with the elevated level of IFN-γ expression. Higher expression of CD26 has been reported in breast carcinoma study [33] while the expression of CD26 level was reduced significantly in the pineapple treatment group. CD26 is expressed by numerous cell types and plays an important role in the immune regulation but due to its unique characteristic, it has distinct roles in different cell type malignancies [33]. Additionally, pineapple vinegar was able to significantly down-regulate TIMP-1 and MMP9. TIMP-1 is a natural inhibitor of MMP9 and involves in the proliferation of cancer cells [34].

In addition, the pineapple vinegar treatment has significantly down-regulated the expressions of IGFBP-1 and IGFBP-2. Studies found that the over expression of IGFBPs level increased the risk of breast cancer through the proliferation process [35, 36]. To note, the level of HGF also decreased significantly in the tumor of PH treated group. HGF promotes the tumor growth by stimulating neovascularization and it was found that the levels of HGF expression in breast cancer patients were elevated as compared to the normal people [37]. Moreover, the level of pentaxin-3 in the tumor of PH treated group decreased significantly. Pentraxin-3 is one of the inflammatory signals induced by cytokines including IL-1β and the increase of pentraxin-3 was found to promote angiogenesis [38]. The expression of MMP-3 decreased significantly in pineapple vinegar treatment group. MMP-3 damages the extracellular matrix inhibitor, deactivates several proteinase inhibitors and increases the proliferation of cancer cells through fibrosis, neovascularization, and tenascin-C expression [39]. Additionally, the level of endoglin decreased significantly in pineapple vinegar treatment group. The over-expression of endoglin in the endothelial cell was found to promote the tumor growth through the proliferation process, increases the invasion and migration of cancer cells and promotes neovascularization [40].

In our previous study, we found that pineapple vinegar was rich in gallic acid (862.61 ± 4.38 μg/mL), caffeic acid (218.91 ± 3.24 μg/mL) and several other phenolic acids. There are many scientific evidences that implied the correlation between the polyphenol derived from fruits and vegetables with the anticancer property [16, 41]. The chemo-preventive effect of pineapple vinegar through the anti-metastasis, anti-inflammatory and apoptosis activities may be strongly related to these bioactive compounds. Previous studies have shown that gallic acid inhibited the progression of several cancers via the suppression of metastasis and inflammation [16, 42–45]. Besides that, caffeic acid was also reported to induce apoptosis and inhibit metastasis and inflammation in previous studies [21, 46]. Thus, the anti-tumor effect of pineapple vinegar might be correlated with the high content of phenolic acids especially gallic and caffeic acid.

## Conclusion

Pineapple vinegar showed an anti-tumor effect on 4 T1 cells in vitro and in vivo via the induction of apoptosis, inhibition of inflammation, suppression of tumor metastasis and activation of immune response. This study reflects a prominent effect of pineapple vinegar as a potential chemo-preventive dietry intervention for breast cancer. As cancer is a group of illnesses consisting various types of diseases [1], future study using other types of cancer cells are needed to evaluate this chemo-preventive effect across different types of cancer.

## Additional file

Additional file 1: **Table S1** Primer sequences. (DOCX 14 kb)

## Data Availability

All relevant data and materials are within the manuscript.

## Abbreviations

CD26: Cluster of differentiation 26
cDNA: Complementary DNA
COX-2: Cyclooxygenase-2
DAB: 3,3′-Diaminobenzidine
ELISA: Enzyme-linked immunosorbent assay
FACs: Fluorescence-activated cell sorter
FBS: Fetal bovine serum
FGF: Fibroblast growth factor
FITC: Fluorescein isothiocyanate
HGF: Hepatocyte growth factor
HRP: Horseradish peroxidase
IACUC: Institutional Animal Care and Use Committees
ICAM-1: Intercellular Adhesion Molecule 1
IFN-γ: Interferon gamma
IGFBP-1: Insulin-like growth factor-binding protein 1
IGFBP-2: Insulin-like growth factor-binding protein 2
IL-10: Interleukin-10
IL-1β: Interleukin-1beta
IL-2: Interleukin-2
iNOS: Inducible Nitric oxide synthases
IP-10: Interferon γ-induced protein 10 kDa
LDH: Lactate dehydrogenase
MDA: Malonaldehyde
MMP-3: Matrix metalloproteinases 3
MMP9: Matrix metalloproteinases 9
MTT: 3-(4,5-Dimethylthiazol-2-yl)-2,5-diphenyltetrazolium bromide
NF-κβ: Nuclear factor kappa-light-chain-enhancer of activated B cells
NH_4_Cl: Ammonium chloride
NO: Nitric oxide
PBS: Phosphate buffer saline
PH: Pineapple vinegar high concentration
PI: Propidium iodide
PL: Pineapple vinegar low concentration
RIPA: Radioimmunoprecipitation assay
RNA: Ribonucleic acid
RT-PCR: Real time polymerase chain reaction
SC: Subcutaneous
SDS: Sodium dodecyl sulfate
TBST: Tris-buffered saline (TBS) and Polysorbate 20
TdT: Terminal deoxynucleotidyl transferase
TIMP 1: Tissue inhibitors of metalloproteinases 1
TUNEL: Terminal deoxynucleotidyl transferase dUTP nick end labeling
UPM: Universiti Putra Malaysia
UT: *in vivo* untreated group
VEGF: Vascular endothelial growth factor
