# Supplementary material for: Apoptosis and metastasis inhibitory potential of pineapple vinegar against mouse mammary gland cells in vitro and in vivo
Source: Nutr Metab (Lond). 2019 Jul 26;16:49. doi: 10.1186/s12986-019-0380-5 (PMC6660685; doi:10.1186/s12986-019-0380-5)
Supplement: Supplementary file 1 — Table S1 Primer sequences. (DOCX 14 kb) [file 12986_2019_380_MOESM1_ESM.docx]

**Table S1 - Primer sequences**

| **Target Genes** | |
| --- | --- |
| **c-MYC** | Forward:5’- TGATGTGGTGTCTGTGGAGAA-3’ |
|  | Reverse:5’- CGTAGTTGTGCTGGTGAGTG-3’ |
| **ICAM-1** | Forward:5’- TGCTCAGGTATCCATCCATCC-3’ |
|  | Reverse:5’- ACGGTGCCACAGTTCTCAA-3’ |
| **iNOS** | Forward:5’-GCACCGAGATTGGAGTTC-3’ |
|  | Reverse:5’-GAGCACAGCCACATTGAT-3’ |
| **NF-κβ** | Forward:5’-CATTCTGACCTTGCCTATCT-3’ |
|  | Reverse:5’-CTGCTGTTCTGTCCATTCT-3’ |
| **Reference Genes** | |
| GAPDH | Forward:5’-TTCCAGCCTTCCTTCTTG-3’ |
|  | Reverse:5’- GGAGCCAGAGCAGTAATC-3’ |
| **β-actin** | Forward:5’-GAAGGTGGTGAAGCAGGCATC-3’ |
|  | Reverse:5’-GAAGGTGGAAGAGTGGGAGTT-3’ |
| **HPRT** | Forward:5’-CGTGATTAGCGATGATGAAC-3’ |
|  | Reverse:5’- AATGTAATCCAGCAGGTCAG-3’ |
